# Neuroeconomic Measures of Social Decision-Making Across the Lifespan

**DOI:** 10.3389/fnins.2012.00128

**Published:** 2012-09-21

**Authors:** Lusha Zhu, Daniel Walsh, Ming Hsu

**Affiliations:** ^1^Virginia Tech Carilion Research Institute, Virginia Polytechnic Institute and State UniversityRoanoke, VA, USA; ^2^Neuroeconomics Laboratory, Haas School of Business, University of California BerkeleyBerkeley, CA, USA; ^3^Helen Wills Neuroscience Institute, University of California BerkeleyBerkeley, CA, USA

**Keywords:** aging, game theory, reinforcement learning, strategic learning, neuroeconomics, decision neuroscience

## Abstract

Social and decision-making deficits are often the first symptoms of a striking number of neurodegenerative disorders associated with aging. These includes not only disorders that directly impact dopamine and basal ganglia, such as Parkinson’s disorder, but also degeneration in which multiple neural pathways are affected over the course of normal aging. The impact of such deficits can be dramatic, as in cases of financial fraud, which disproportionately affect the elderly. Unlike memory and motor impairments, however, which are readily recognized as symptoms of more serious underlying neurological conditions, social and decision-making deficits often do not elicit comparable concern in the elderly. Furthermore, few behavioral measures exist to quantify these deficits, due in part to our limited knowledge of the core cognitive components or their neurobiological substrates. Here we probe age-related differences in decision-making using a game theory paradigm previously shown to dissociate contributions of basal ganglia and prefrontal regions to behavior. Combined with computational modeling, we provide evidence that age-related changes in elderly participants are driven primarily by an over-reliance in trial-and-error reinforcement learning that does not take into account the strategic context, which may underlie cognitive deficits that contribute to social vulnerability in elderly individuals.

## Introduction

A widow responds to a telemarketing investment firm’s offer of financial security. The firm convinces her to convert all her assets to risky, liquid investments managed by the firm. Over the course of a year, the firm provides near constant attention to the widow, who, by the end of the year, had lost $800,000 (Starnes, 1996). Such crimes are unfortunately common. Although there is widespread recognition of elderly fraud among both financial and legal scholars, and efforts to introduce legislation to combat this problem (e.g., Smith, 2000), we know very little about the specific sources of such vulnerability at the neurobiological level. Unlike memory and motor impairments, which are readily recognized as symptoms of more serious underlying neurological conditions, decision-making deficits often do not elicit comparable concern in the elderly (Denburg et al., 2007). There are also few neuropsychological tools or biomarkers available to measure decision-making deficits, particularly those that contain a social component such as susceptibility to fraud.

Here we sought to probe age-related effects of an important class of social behavior captured by economic games, and build upon recent advances in understanding of the neural substrates of value-based decision-making. Intuitively, efficient value-based decision-making requires organisms to make decisions to obtain rewards and avoid punishments that are present in the environment (Fehr and Camerer, 2007; Rangel et al., 2008; Maia and Frank, 2011). In the social domain, however, organisms also need to anticipate and respond to actions of others competing or cooperating for the same rewards.

Neurobiologically, there is much evidence that the capacity to make appropriate value-based decisions depends critically upon integrity of the nigrostriatal dopamininergic (DA) system and frontostriatal circuits, which is well known to degenerate over the course of aging (Bäckman and Farde, 2005). Furthermore, there is growing consensus that the computational underpinnings of these systems can be parsimoniously characterized by reinforcement learning (RL) theories of behavior (Sutton and Barto, 1981; Schultz et al., 1997). This synthesis of theory and data has led to speculations that abnormalities observed in healthy older adults is at least partially caused by age-related decreases in neuronal number in these circuits, as well as a decreased number of synapses in those neurons (Li et al., 2001; Li and Sikström, 2002; Samanez-Larkin et al., 2007).

Despite this rapid progress, however, there has been limited application of this formal framework to understand age-related changes in value-based decision-making in the social domain. Here, in addition to needing to learn about available rewards and punishments in the environment, agents also need to anticipate and respond to cooperative or competitive actions of others (Camerer, 2003; Lee, 2008). This requires the ability to behave strategically, which has been the subject of intense study in theoretical biology and game theory (Fudenberg and Levine, 1998; Hofbauer and Sigmund, 1998). Game theory provides a mathematically precise description of the social environment, thus allowing for quantitative modeling of behavior that can build upon previous findings on reward learning (Fehr and Camerer, 2007; Lee, 2008).

An important insight of this literature is that standard RL models provide an incomplete account of strategic learning. Individuals blindly exhibiting RL behavior in social and strategic settings are essentially ignoring the fact that their behavior can be exploited by others (Camerer, 2003; Hampton et al., 2008). In contrast, another well-studied class of learning models, commonly referred to as belief-based learning, requires players to form and update first-order beliefs regarding the likelihood of future actions of opponents through experience, and provides a tractable model of social learning in relatively simple environments. Neurobiologically, there is converging evidence that social decision-making depends upon a broader network of regions that project to the striatum, in particular the medial prefrontal cortex (mPFC), which is widely thought to be intimately involved in “theory of mind” critical for social cognition and strategic reasoning (Amodio and Frith, 2006; Jackson et al., 2006; Saxe, 2006).

Results from our previous research has shown that this paradigm engaged key components of the frontostriatal circuits, and to dissociate their respective contributions to behavior (Zhu et al., 2012). Specifically, using model-based fMRI, activity in the ventral striatum was found to underlie standard model-free RL through trial and error. In contrast, activity in the mPFC underlies more cognitively sophisticated belief learning that involves forming and responding to first-order beliefs about the actions of other individuals. Based on these results, we hypothesize that the ability to make advantageous social decisions would decline over age as a result of decline in higher-order cognitive functions that we believe to be crucial for complex social decision-making. Furthermore, we hypothesized that the differences in behavior can be captured by key parameters in the computational model across age cohorts.

## Materials and Methods

### Subjects

We compare results from 30 young subjects (16 female, mean age 23.3 ± 4.6 years) from University of Illinois at Urbana-Champaign, and 29 elderly subjects (14 female, mean age 64.1 ± 5.4 years) recruited from: (1) local flyers and bulletins in the Berkeley community, (2) online forums such as Craigslist, and (3) Berkeley Retirement Center (Table 1). All elderly subjects were tested on the mini-mental status exam and self-reported to be healthy and with no significant neurological issues.

**Table 1:** **Demographic information of participants**.

Age group	Young	Elderly
Mean age	23.3	64.1
(S.D)	(4.6)	(5.4)
*N*	30	29
(# Female)	(16)	(17)
Mean year education	14.4	15.0
(S.D)	(1.1)	(0.9)
Estimated WAIS-R IQ		109
(S.D)		(9.2)
WCST% correct		68.3
(S.D.)		(14.4)
WCST% perseverative errors		11.7
(S.D)		(6.9)

### Experimental paradigm

We used the “Patent Race” game, first studied experimentally by Rapoport and Amaldoss (2000), and most recently used in our previous neuroimaging study. This game is simple in motivation but rich in the strategic nuances and the patterns of behavior that it can generate (Zhu et al., 2012). In the game, two opposing players are randomly matched from a large pool of players at the beginning of each round and compete for a prize by choosing how much to invest (in integer amounts) from their respective endowments. The player who invests more wins the prize, while the other loses. In the event of a tie, neither player wins the prize. Players keep the part of their endowment that is not invested.

**Figure 1:**
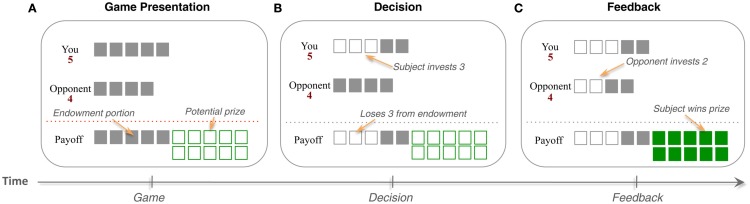
**Patent Race Game. (A)** Two players are randomly matched from a large pool of players at the beginning of each round and **(B)** compete for a prize by choosing an investment (in integer amounts) from their respective endowments. **(C)** The player who invests more wins the prize and the other loses. In the event of a tie, both lose the prize. Players keep the part of their endowment that is not invested. In the particular payoff structure of this game, the prize size is 10, and players are of two types: Strong and Weak. The Strong player has five units to invest, whereas the Weak player has four units to invest.

In the particular payoff structure that we use, the prize size is 10, and players are of two types: *Strong* and *Weak*. The Strong player has five units of endowment, and can invest between 0 and 5 units in integer amounts, whereas the Weak player has four units to invest, and can invest between 0 and 4 units (Figure 1). Furthermore, to reduce cognitive burden associated with playing this relatively complex game, we used a new interface first introduced in Zhu et al. (2012). This interface replaced the standard matrix form representation of the game that contains 60 elements with one that directly reflects the logic of the game.

### Procedure

Upon arrival at the laboratory, subjects were given instructions and quiz to ensure the understanding of the experiment. Participants played two stages of 80 rounds each of strong and weak roles (counterbalanced). Opponents’ choices were drawn from a pool of 16 young adults who participated in an earlier session at University of Illinois at Urbana-Champaign. We ran subjects separately to allow us to pair players against a common distribution of opponents. Previous sessions comparing “live” sessions and “non-live” sessions show that young adults do not differ significantly across treatments (Zhu et al., 2012). All subjects were fully informed of the purposes of the research and were free to withdraw without penalty. Elderly participants further completed psychometric tests for IQ (Shipley Institute of Living Scale) and executive functioning (Wisconsin Card Sorting Task, WCST).

### Computational modeling

To quantitatively compute the mapping from the stimulus inputs to the behavioral observations, we used the “experience-weighted average” (EWA) model first introduced by Camerer and Ho (1999). This model embeds both RL and belief learning, two of the most widely used approaches to studying learning in competitive games.

These two learning rules differ with regards to the information that subjects use to update action values. Intuitively, at the end of each round, the subject receives two pieces of information – the received rewards in the form of payoffs, and how much the opponent invested. For example, consider two rounds where the subject chose 5, but where the opponent chose 0 in one round and 4 in the other. In both cases, the subject’s received payoff is 10. However, in the former the subject could have earned more by investing less, with the optimal investment being 1. In the latter case, however, the subject cannot improve by investing any other amount.

Under RL, players are assumed to ignore the actions of the opponent, and as a result treat both cases as equivalent. On the other hand, belief learning assumes that players, either directly or indirectly depending on the particular interpretation, include this information in updating of action values (Cheung and Friedman, 1997; Camerer, 2003). The hybrid EWA model provides a parametric account of the weighting between the two learning rules, as well as capturing how the past experiences depreciate over time, both of which we will study in our data.

Formally, on each round, player *i* assigns a value $V_{\text{i}}^{\text{k}}\left(t\right),$ to each strategy $S_{\text{i}}^{\text{k}}$ in the strategy set $S_{\text{i}}=\left\{S_{\text{i}}^{\text{1}},\;S_{\text{i}}^{\text{2}},\;\ldots ,\;S_{\text{i}}^{\text{k}\;}\right\},$ (i.e., investment amount). They also come into the game with certain prior beliefs *N*(0), which reflect either the result of logical deduction or previous life experiences. Denote *S*_*i*_(*t*) as the investment amount by player *i* at period *t*, and *s*_−*i*_(*t*) as the investment amount of the opponent at period *t*, the evolution of $V_{\text{i}}^{\text{k}}\left(t\right)$ and *N*(*t*) is governed by three parameters and updates according to the following:

$$\begin{array}{l} V_{\text{i}}^{\text{k}}\left(t\right)=\left\{\begin{array}{c} \frac{\phi_{\text{i}}N\left(t-1\right)V_{\text{i}}^{\text{k}}\left(t-1\right)\;+\;\delta_{\text{i}}\pi_{\text{i}}\left(S_{\text{i}}^{\text{k}},\;S_{-\text{i}}\left(t\right)\right)}{N\left(t\right)},\;\;\text{if}\;S_{\text{i}}^{\text{k}}\neq S_{\text{i}}\left(t\right)\\ \frac{\phi_{i}N\left(t-1\right)V_{\text{i}}^{\text{k}}\left(t-1\right)\;+\;\pi_{\text{i}}\left(S_{i}^{\text{k}},\;S_{-\text{i}}\left(t\right)\right)}{N\left(t\right)},\;\;\text{if}\;S_{\text{i}}^{\text{k}}=S_{\text{i}}\left(t\right)\\ \; \end{array}\right.\\ N\left(t\right)=\rho_{\text{i}}N\left(t-1\right)+\;1 \end{array}$$

As discussed in Camerer and Ho (1999), the three parameters capture qualitatively distinct aspects of the learning process. First, two of the parameters describe distinct notions of “experience”: pre-game experience (or prior beliefs) and in-game experience. Updating of the former (pre-game prior beliefs) is controlled by the parameter ρ_*i*_, such that a large value of ρ_*i*_ leads prior beliefs to wear off quickly. On the other hand, updating of in-game adaptation – that is, responsiveness to actual experience during the game – is captured via the parameter Φ_*i*_, where smaller values imply greater weight placed on recent game experience. Finally, the weight between reinforcement and belief learning is captured by the parameter δ_*i*_, which reduces to pure RL when δ_*i*_=0, and to pure belief learning model when δ_*i*_=1.

To convert latent values $V_{\text{i}}^{\text{k}}\left(t\right)$ to choice probabilities, we assume that the probability of player *i* playing $S_{\text{i}}^{\text{k}}$ follows a softmax distribution $P_{i}^{k}\left(t-1\right)=\exp\left(\lambda ∙v_{i}^{k}\left(t\right)\right)∕\sum_{\text{l = 1}}^{\text{L}}\exp\left(\lambda ∙v_{i}^{k}\left(t\right)\right),$ where λ is a measurement of subjects’ sensitivity to differences in latent values (Camerer and Ho, 1999; Hsu et al., 2005). Using initial values *N*(0) and $V_{\text{i}}^{\text{k}}\left(t\right)$ calculated from first period data (Roth and Erev, 1995; Ho et al., 2008), we performed maximum likelihood estimation at the individual level for both young and elderly cohorts using a grid search over a large range of values for all free parameters. That is, we maximized for each subject the log-likelihood function $\sum_{\text{t}}\log\left(P_{\text{i}}^{{\text{s}}_{\text{i}}\left(t\right)}\left(t\right)\right)$ Standard errors were estimated through a jackknife procedure (Camerer and Ho, 1999; Zhu et al., 2012).

## Results

Our primary hypothesis is that elderly adults will exhibit slower adaptation in strategic learning as compared to young adults. That is, elderly adults will be less responsive to the actions of opponents in terms of choice behavior. Furthermore, using our computational paradigm, we aim to distinguish between contributions of three non-mutually exclusive computational accounts of any observed age-related changes. First, we test whether older adults employ less belief-based learning, and rely more upon simpler RL. This would suggest that behavioral differences are caused by not taking a complete account of possible information in the decision context. Second, we test the hypothesis that older adults may be less sensitive to recent in-game experiences. This will be reflected in the estimated values for parameter Φ, and can intuitively capture the notion that older adults are more “sluggish” in their adjustment process (Kovalchik et al., 2005). Third, we test whether older adults exhibit stronger pre-game prior beliefs, captured by the parameter ρ, which would suggest that they are more “stubborn” in the sense that their pre-game prior belief decays slower. These hypotheses and a discussion of the different parameters are summarized in Table 3.

### Model-free Measures

We begin with simple model-free comparisons of choice behavior across age groups. Table 2 presents the empirical frequencies of choices for each age group separated by player role. In order to provide a benchmark for such comparison, we also include Nash equilibrium choice probability predictions. The unique Nash equilibrium prediction is that strong players should invest five 60% of the time, one and three 20% of the time respectively, and weak players invest zero 60% of the time, two or four 20% of the time. As shown in Table 2, young subjects on average were reasonably close to the Nash equilibrium prediction with the exception of overinvesting 4 and underinvesting 3 as strong players, whereas the distribution of choices made by elderly strong subjects were further from Nash equilibrium prediction, with more evenly distributed choice over investing 2, 3, 4, and 5. Yet as weak players, both elderly and young subjects overinvested 0 and underinvested 4. However, elderly subjects also overinvested 1, which is the iteratively dominated strategy for the weak role. A test of the proportion of deviation showed that play from elderly cohort’s deviation from Nash equilibrium significant more than did the young cohort (*p* < 0.01).

**Table 2:** **Comparison of Nash equilibrium prediction with the empirical frequencies from young and elderly cohorts**.

Role	Investment	Equilibrium prediction (%)	Empirical distribution	
			Young (%)	Elderly (%)
Strong	0	0	1	1
	1	20	18	12
	2	0	10	16
	3	20	11	29
	4	0	16	22
	5	40	45	21
Weak	0	40	49	39
	1	0	3	13
	2	20	6	7
	3	0	13	11
	4	20	27	30

**Figure 2:**
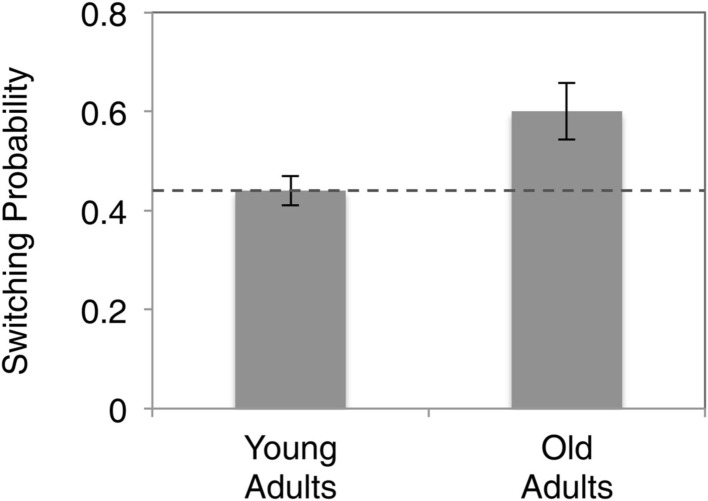
**Comparison of probabilities of “staying” across different age groups**. Dashed line indicates Nash equilibrium predicted probability of repeating the same investment.

To examine the “stickiness” of choices between successive rounds, we computed the instances where participants switched investment levels versus those where they did not. This gives us an index of the proportion of rounds in which participants switched strategies, versus those rounds in which they stayed (Figure 2). We found that young subjects on average repeated investment in 44% of the choices over the course of the experiment, which is remarkably similar to the Nash equilibrium prediction. In contrast, we found that elderly subjects repeated previous investments at a much higher rate (60%), and significantly greater than more often than young adults (*p* < 0.05).

### Model-based measures

To provide a mechanistic account of the differences as measured using the model-free measures, we next fitted choice behavior of our participants using the EWA model. First, we compared the mean goodness of fit of the model across both cohorts. A significant difference may suggest that comparisons of estimated parameters are biased due to different explanatory powers of the model. We found, however, that the mean log-likelihood values did not differ significantly between the young and old cohorts (Table 3, *p* > 0.2). This suggests that our computational model is able to capture trial-by-trial variations in behavior at a similar rate for both cohorts.

**Table 3:** **EWA parameters and their interpretations, as well as parameter estimates from young and old cohorts**.

Model estimates	Parameter interpretation	Young adults^1^	Old adults^1^	*T*-test *p*-value^2^	K–S test *p*-value^2^
*LL*	Log-likelihood value	90.4 (5.67)	79.3 (8.26)	0.27	0.56
δ	Degree of belief-based learning exhibited. Larger values mean more belief-based learning.	0.48 (0.041)	0.28 (0.075)	0.022 (0.065)	0.0016 (0.0047)
ρ	Depreciation of the strength of before-game prior beliefs. Larger values mean more depreciation.	0.86 (0.043)	0.83 (0.068)	0.74 (1.0)	0.59 (1.0)
Φ	Weight placed on most recent experience. Smaller values mean more weight on freshest experience.	0.88 (0.031)	0.85 (0.050)	0.53 (1.0)	0.57 (1.0)

*^1^Parentheses indicate SEM*.

*^2^Both *t*-test and Kolmogorov–Smirnov test *p*-values are given, with those corrected for multiple comparisons (three tests) given in parentheses*.

Next, we compared the mean values of the individual-level parameter estimates (Table 3). We found that the mean estimates for parameter δ was significantly lower for the elderly as compared to the young (0.48 for young, 0.28 for elderly, *p* < 0.05), indicating that the elderly on average employ less belief-based strategy, and more reinforcement. This is in line with the findings through fMRI that there may exist significant tissue loss in gray matter volume in mPFC, which is indicated to be involved in belief-based learning in our previous study (Zhu et al., 2012).

In contrast, we found that mean estimates for both types of discount rates did not differ significantly between young and elderly cohorts. Both young and elderly cohorts were estimated to have a similar value of Φ (0.95 for the young; 0.89 for the elderly, *p* > 0.1), suggesting that both groups responded smoothly to past in-game experience. Similarly, both groups also discounted prior-game beliefs, captured by parameter ρ, at approximately similar levels (*p* > 0.1, Table 3). Neither finding can be explained by differences in the learning environment as both cohorts faced the same pool of opponents.

**Figure 3:**
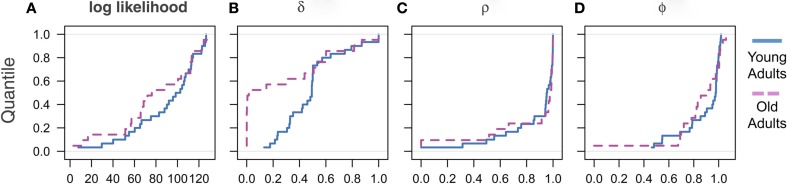
**Empirical cumulative distributions of estimated parameters across cohorts. (A)** Log likelihood values measuring degree of model fit, **(B)** parameter δ capturing the degree of belief learning, **(C)** parameter ρ measuring depreciation of the strength of before-game prior beliefs, and **(D)** parameter Φ measuring weight placed on most recent experience.

Motivated by findings in the aging literature that aging increases variability of behavioral responses (Samanez-Larkin et al., 2010), we next investigated individual differences in the learning parameters using our model. We therefore compared the empirical cumulative distributions of the model fit and parameter estimates across the cohorts. We found that the distribution of the model fits of the two cohorts, as measured by the log-likelihood value, is distributed similarly, such that there is no indication of increased variance or clustering of the elderly cohort (Figure 3A). Similarly, we found that the two discounting parameters are also similarly distributed across age cohorts. That is, there was no indication of increased variance in either of the discounting parameter estimates (Figures 3C,D).

In contrast, the mediation parameter δ showed that behavior in approximately half of the elderly cohort was driven entirely by RL (Figure 3B). In light of the non-normal distribution of the δ parameter estimates, we also included significance tests using the Kolmogorov–Smirnov (K–S) test, a non-parametric, distribution-free test that is robust to violations of normality (Table 3). We found that all results using the K–S test are consistent with those using the *t*-test, and show highly significant differences in the δ parameters between age cohorts (*p* < 0.001). Interestingly, the remaining elderly cohort appear to be distributed similarly to the young adults, as can be seen by the upper half of the distribution (Figure 3B). There was, however, no indication that this group of pure reinforcement learners differed on other dimensions. Surprisingly, we found no differences in either demographic or other model estimates for these two groups. The only measure that approached significance was the value of ρ. The low δ group had a slightly higher mean ρ (0.95) compared to the high δ group (0.70), which is significant at the *p* < 0.1 level. This lack of differentiation, however, may well be due to a lack of power in our sample given the relatively modest sample size and restricted range of age for the elderly cohort.

## Discussion

We spend much of our lives devoted to the accumulation of financial and social prosperity, and often with much success. To take just one measure, the median net worth of a 65-year-old American in 2007 is more than double that of a 40 year old (Bucks et al., 2009). For many, however, such wealth comes at a vulnerable time when the cognitive and neurological apparatus that made this possible is beginning to break down (Plassman et al., 2008). This vulnerability can be attributed in part to a decline in the ability to make decisions that take into account the appropriate cost-benefit tradeoffs. Often these decisions take on a social dimension, where the elderly appear particularly vulnerable. For example, it is well known that the elderly are disproportionate targets of fraud across the world, and constitute a conservatively estimated 30% of all fraud victims in the United States (Templeton and Kirkman, 2007; Bucks et al., 2009).

An understanding of the neurocognitive substrates of these vulnerabilities therefore depends upon the availability of neuropsychological tools that can be used to probe and characterize such decision-making deficits, particularly those that contain a social component such as susceptibility to fraud. This work makes two contributions toward this goal. First, we show our novel social learning paradigm was able to probe behavioral differences between cohorts, as well as individual differences within cohorts. We found that, in contrast to young adults, learning in about half of the elderly adults is driven primarily by RL. Future studies can explore the degree to which such changes are present in other social and non-social settings that require higher-order cognition, such as cooperative interactions (King-Casas et al., 2005; Chang et al., 2011), or those involving explicit task structure (Ribas-Fernandes et al., 2011; Simon and Daw, 2011). Second, using a well-established computational model of strategic learning, we were able to dissociate between two possible sources of the observed differences. Previous accounts have largely focused on qualitative descriptions of “sluggishness” of adjustment of behavior. However, such a behavior can be a result of either (1) an inability to integrate new information into one’s reward expectations by discounting previous experiences, captured by the two different discounting parameters, or (2) an attenuation of the ability to extract or integrate information beyond the received rewards and punishments. We show that the behavior is primarily driven by the latter, and in particular an attenuation of the ability to observe or integrate actions of others or counterfactual outcomes. In contrast, elderly individuals did not show significant differences in two types of discounting of past experiences.

More broadly, our results potentially shed light on contradictory findings in previous psychological and economic studies of age-related effects in relation to social behavior. In particular, a number of studies have suggested a decline in the ability of theory of mind with normal aging (McKinnon and Moscovitch, 2007; Slessor et al., 2007). Yet others found that older adults actually performed better than younger adults, even in the face of possible decline in many forms of cognitive processing (Happé et al., 1998; Grossmann et al., 2010). Using a behavioral economic approach similar to ours, Kovalchik et al. (2005) compared the ability of strategic reasoning between the young and healthy elderly subjects using the so-called “*p*-beauty contest.” This task has been widely used in previous behavioral studies using traditional undergraduate subjects as well as non-standard populations such as business executives and portfolio managers (Nagel, 1995; Duffy and Nagel, 1997). Surprisingly, they similarly found no significant difference between the healthy elderly (mean age 82) and young undergraduate participants.

Combined with our results, however, these results suggests that the diminished reliance on mentalization and/or counterfactual information to *dynamically update* behavior may reflect core changes in the cognitive processing of social information that occurs over the aging process. This is as opposed to strategic reasoning, which refers to the *static* inferential process of guessing what others will do without any prior contractual agreement, which may well be preserved during aging. This hypothesis is consistent with previous findings of behavioral deficits in elderly patients in the “Iowa Gambling Task” (IGT; Bechara et al., 2000), as well with what is known about degeneration of the dopaminergic circuits and mPFC that supports value-based decision-making. In particular, longitudinal studies have found frontal lobes suffered the most drastic loss of volume as assessed through MRI (Resnick et al., 2003).

In contrast, we speculate that static reasoning capacities in the elderly may be partially preserved by reallocation of processing resources from other brain regions. Such compensatory processes at the neural level have been found across a variety of cognitive functions, including episodic retrieval and visual perceptual attention, and which occur even in the face of global declines in neural integrity (Davis et al., 2008). For example, there is abundant evidence that older adults compensate for declines in bottom-up sensory processing by over-recruitment of top-down processes mediated by PFC (Davis et al., 2008; Dennis and Cabeza, 2008).

In the case of social cognitive functioning, there is substantial evidence that, during development, the so-called “mentalizing system” – consisting of the anterior mPFC, the posterior superior temporal sulcus at the temporoparietal junction (pSTS/TPJ), and the anterior temporal lobe (ATL) – undergo substantial changes in their functional response to social information such as mental states (Paus, 2005; Blakemore, 2008; Burnett et al., 2009). Adolescents have been shown, for example, to exhibit greater activity within the mPFC than do adults in social cognition tasks (Burnett et al., 2009). In contrast, we know much less about how neural responses change over adulthood in the social cognition and behavior (Castelli et al., 2010; Beadle et al., 2012; Moran et al., 2012), and whether they might have compensatory functions that have been documented for other cognitive functions. A more complete account of these age-related changes, however, is only possible with a proper characterization of the computational and structural integrity of the underlying neural systems and their interactions.

## Conflict of Interest Statement

The authors declare that the research was conducted in the absence of any commercial or financial relationships that could be construed as a potential conflict of interest.
